# The effects of mental fatigue on sport-specific motor performance among team sport athletes: A systematic scoping review

**DOI:** 10.3389/fpsyg.2023.1143618

**Published:** 2023-04-11

**Authors:** Rui Yuan, He Sun, Kim Geok Soh, Alireza Mohammadi, Zakaria Toumi, Zhendong Zhang

**Affiliations:** ^1^Physical Education Department, University of Shanghai for Science and Technology, Shanghai, China; ^2^School of Physical Education, Zhengzhou University, Zhengzhou, China; ^3^Department of Sport Studies, Faculty of Education Studies, Universiti Putra Malaysia, Selangor, Malaysia; ^4^Faculty of Business Management, City University Malaysia, Selangor, Malaysia; ^5^Department of Human Anatomy, Faculty of Medicine and Health Sciences, Universiti Putra Malaysia, Selangor, Malaysia

**Keywords:** team sport, motor performance, athletes, mental fatigue, scoping review

## Abstract

**Background:**

The psychobiological state known as mental fatigue (MF) is by engaging in mentally taxing activities for an extended period, which is typically found in team sports, of the high cognitive demand and unpredictable environment. It increases the perception of effort and influences executive functions, impairing sport-specific performance in athletes. However, the consequences of MF on sport-specific motor performance (SSMP) among athletes in team sports remain unclear.

**Objective:**

This scoping review seeks to find and map research publications that investigate the effect of MF on SSMP in team sports.

**Methods:**

Web of Science, Scopus, and PubMed were searched as the main databases, and CENTRAL, Psychology, and Behavioral Sciences Collection, SPORTDicus obtained from EBSCOhost, as well as gray literature was searched for relevant literature and Google Scholar. Cognitive tasks before the SSMP exam are the focus of the selected literature on mental exhaustion. Only experiments testing mental and non-mental exhaustion were chosen.

**Results:**

Twelve studies fulfill the requirement of selection criteria. SSMP in team sports, including soccer, basketball, cricket, and Australian football mainly is examined as physical and technical performance. More specifically, MF significantly influenced physical performance measured as intermittent endurance and total distance (*P* < 0.05), while data was inclusive when assess in an ecological setting (e.g., small-sided game) (*P* > 0.05). Technical performance was mainly measured as ball loss, errors in passing and shooting, interception, and successful tackle and showed a dramatic impairment (*P* < 0.05). The decline of physical activity is relevant with higher level PRE, while decreased technical performance is related to impaired attention resources shown as visual perceptual.

**Conclusion:**

MF adversely influences SSMP in team sports. The most relevant theory for future study to examine the impacts of MF on team-sport athletes could be the psychological model of exercise and its potential extension on attention resources, rather than the traditional “catastrophe” theory.

## Introduction

Team sports refer to sports in that athletes are organized together and played by teams that move freely across a field and score by manipulating a ball into predefined goals (Cockburn et al., 2013). In this context, Fatigue is characterized by a drop in force or power related to prolonged exertion and a decline in performance (Reilly, 1994). Based on these data, it is believed that a performance decline during a match is symptomatic of an athlete's acute physiological impairment and acute fatigue (Mohr et al., 2003, 2005).

These physiological impairments were well explained by the so-called “catastrophe” theory (Noakes and St Clair Gibson, 2004; Noakes et al., 2005) in the team sports context, which has dominated the literature, whereby mechanisms (either, or a combination, of peripheral and central factors) restrict muscular contraction, decreasing physical capacity (e.g., stamina and power) (Bangsbo et al., 2006). However, as another form of fatigue, MF has attracted more and more attention recently (Sun et al., 2021, 2022e; Skala and Zemková, 2022), and it seems unrelated to physiological changes (Marcora et al., 2009). Moreover, it is cognitive from brain activities and associated with reduced sports performance, especially at the end of the matches (Smith et al., 2018). Therefore, to have an overall picture of the psychophysiological indicators underpinning existing theory that explains reduced performance in team sports with mentally fatigued athletes, and provide insight for future studies, there is the necessity to have a review.

Notably, cognitive demands in team sports are exceptionally difficult (Faubert and Sidebottom, 2012; Heppe et al., 2016). That is, neither your teammates nor your opponents can predict the paths that their movements and the ball will take; disruptions can cause a change in direction, and a variety of occlusions in which objects partially or completely disappear from vision. As a result, athletes must be alert all game long (Faubert and Sidebottom, 2012; Smith et al., 2018) and face high demands of cognitive challenge, which highly increases the likelihood of MF. Probably because of this challenge, current evidence has only focused on cognitive performance in team sports. For example, Skala and Zemková (2022) summarized 12 studies and found that MF significantly influences cognitive performance such as decision-making skills, perception, and attention abilities with different durations and intensity of prior mental exertion. Similar findings were detected by Dong et al. (2022). Notably, in team sports, SSMP is critical since it determines a team's success or failure by allowing them to perform skills such as passing, shooting, and tackling (Sun et al., 2021) and move efficiently to create scoring chances (Silva D. C. D. et al., 2022). Therefore, a synthesis with SSMP is necessary for mentally fatigued athletes.

As a type of acute fatigue, MF is a psychobiological state brought on by sustained mental work and manifested as a general sense of weariness and lethargy (Boksem and Tops, 2008; Marcora et al., 2009). MF has been demonstrated to impede physical performance in healthy individuals, such as distance-clamped self-paced running (MacMahon et al., 2014; Pageaux et al., 2014), muscle endurance (Pageaux et al., 2013), and maximal voluntary contraction (Martin et al., 2015; Pageaux et al., 2015).

In sports science, Marcora et al. (2009) introduced an emphasis on the influence of MF on physical performance (e.g., endurance) almost a decade ago. They observed that MF worsened cycling time to exhaustion. Surprisingly, none of the observed physiological indicators (e.g., heart rate, oxygen uptake, and the concentration of blood lactate) altered in conjunction with this performance decline. The only factor linked with the decline in physical performance was a higher evaluation of the rating perception of effort (RPE). In addition, Martin et al. (2018) found that MF reduced dopamine levels in the ACC, which may explain why RPE levels changed in those individuals. Notably, This ACC activation also may lead to impaired executive functions, such as decreased movement monitoring (Lorist et al., 2005; Boksem et al., 2006), response time or accuracy (Lorist et al., 2005), which are highly associated with technical performance among team sports (Sun et al., 2021).

Several narrative (Habay et al., 2021; Sun et al., 2021; Cao et al., 2022) and quantitative (Clemente et al., 2021; Grgic et al., 2022) attempts to synthesize the literature have been made in recent years, whereby performing prior cognitive tasks has led to subsequent decreases in sports performance. Nevertheless, they did not focus on the impacts of MF on SSMP, probably because the condition of MF is in the cognitive domain and consistently it's considered to hinder cognitive performance in sports.

Notably, in a sport-specific context, SSMP refers to the explicit physical actions or the outcomes of a task (Schmidt and Lee, 2005). In terms of SSMP outcomes, common indicators encompass physical (e.g., endurance, power, etc.) and technical performance (e.g., passing, dribbling, etc.). Therefore, the present scoping review aimed to map: (i) research article addressing the impacts of MF on SSMP in team sports; (ii) understand existing theories about fatigue that explained reduced performance in the athletes of team sports context; and (iii) identify literature gaps and make suggestions for further research.

## Methods

The methods and analysis strategy for this scoping review were documented in the prospective registry for systematic reviews (INPLASY: ref. INPLASY202250039). Protocols on the consequences of mental tiredness can be found in INPLASY, but none of them specifically address SSMP, especially in team sports. By this means, the novelty of the suggested protocol was ensured.

We conducted the review to map our important findings addressing the impact of MF on SSMP in team sports with the following 5 stages proposed by Arksey and O'Malley (2005) and Levac et al. (2010); (i) the identification of the research questions; (ii) the identification of the relevant studies; (iii) the selection of the studies; (iv) charting the data; and (v) summarizing and reporting the results.

In this study, we aimed to address three specific research questions:

(1) What are the impacts of MF on SSMP regarding physical and technical performance?
(2) How to understand existing theory related to performance reduction in team sports context with the condition of MF?

### The identification of relevant studies

With the assistance of a medical librarian, a literature search was conducted between March 2022 and May 2022 with no date/year restrictions, searching for the key terms in the following databases: Web of Science, PubMed, Scopus, EBSCOhost (CENTRAL, Psychology and Behavioral Sciences Collection, SPORTDicus), and. The Boolean search query used for our databases search encompassed “mental fatigue” and its alternative terms, combined with the varied terms of SSMP such as physical performance and technical performance; specifically, they are (“mental fatigue” OR “mental exertion” OR “cognitive fatigue” OR “cognitive exertion” OR “mental exhaustion” OR “mental tiredness”) AND (sport) AND (“technical performance” OR “technique” OR “skill” OR “physical performance” OR “endurance” OR “power” OR “performance”). The details of the search strategy were shown in Supplementary Table S1.

### The selection of relevant studies

**Figure 1:**
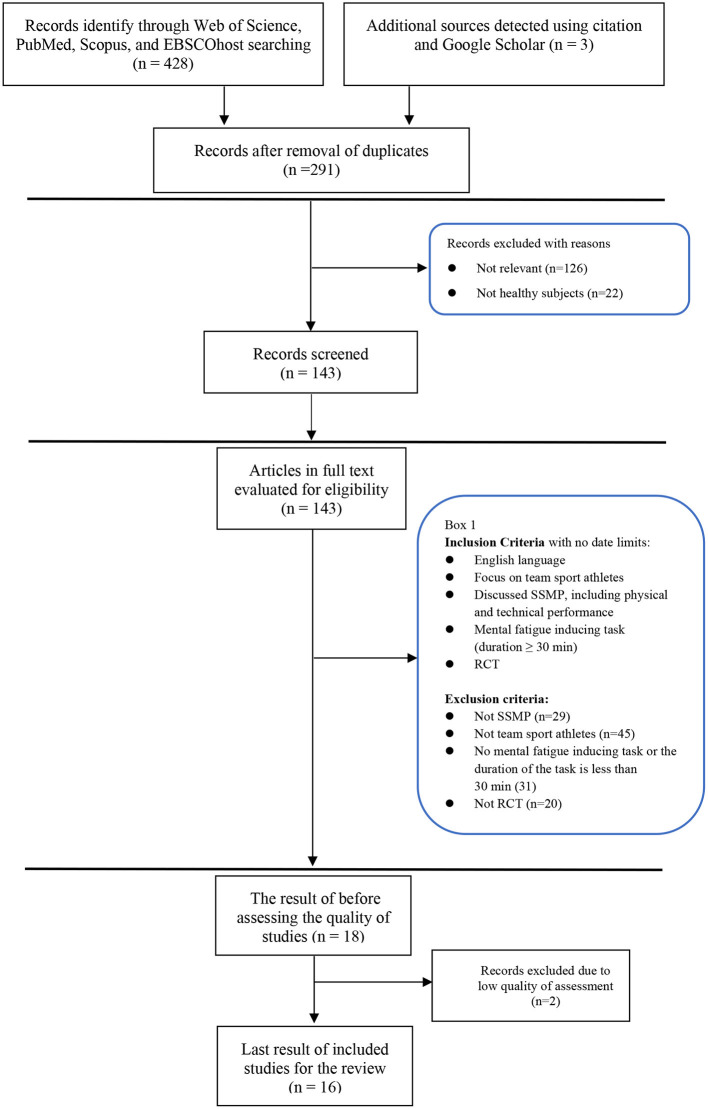
PRISMA summary of the study selection process.

As directed by the Joanna Briggs Institute, Figure 1 depicts the research team's search and selection procedure (Peters et al., 2015). The current review considered the following criteria to retrieve the relevant studies. Specifically, the studies were included, if they examined:

(i) a variety of levels of athletes without any injury such as amateur, semi-professional, and professional;
(ii) athletes are organized together and played by teams that move freely across a field and score by manipulating a ball into predefined goals;
(iii) with a task for prior mental exertion;
(iv) any performance tasks measured SSMP;
(v) with a design of a randomized controlled trial;
(vi) with an English publication.

Initial identification yielded 431 articles. S.H. and A.M. assessed study titles for the inclusion of abstracts. The abstracts were then divided between the researchers A.M. and S.K.G., while S.H. assessed all abstracts (*n* = 291) separately. S.H. reviewed with each researcher their independent evaluation of the abstracts to determine eligibility for full-text review based on the inclusion criteria (Figure 1: Box 1). This led to the identification of 143 publications that were thoroughly evaluated by all scholars. All researchers discussed any disparities until an agreement was reached. Then, it was determined that 18 papers met the inclusion requirements.

However, two studies (Kunrath et al., 2018; Bahrami et al., 2020) identified low quality through the quality assessment with “QualSyst”, including 14 items shown in Supplementary Table S2. The score (no = 0, partial = 1, yes = 2) is determined by the degree to which a particular requirement was met. The letter “NA” denotes items that were removed from the calculation of the total score because they were irrelevant to the study design. The summary score for each study was derived by tallying all the points achieved and dividing that number by the highest possible score. The scores of 55%, 55–75%, and 75% reflect low, medium, and high quality, respectively. Any low-quality studies should be omitted (Kmet et al., 2004). Finally, 16 studies were included in the review.

### Charting the data

A descriptive analysis method was applied to the 16 publications found through the selection procedure depicted in Figure 1. The characteristics of each article were summarized in an Excel spreadsheet, including publication year, study location, author, research purpose, study type, data collection techniques, a summary of results, and important messages.

### Summarizing and reporting results

The final component of the framework is a summary of the selected studies (e.g., Arksey and O'Malley, 2005). As the aim of this systematic scoping review was to identify studies that presented studies about the effects of MF on SSMP among team sport athletes, our reporting on the key message such as prior mentally fatiguing tasks, SSMP, and outcome assessment, including physiological, and psychological changes from each study. To enhance the understanding, some specific statistics were reported in the review; they are: mean differences between experimental and control groups; 90 and 95% confidence interval; and effect size (Cohens d ES [d]) or Partial eta squared ES [$\eta_{p}^{\;2}$]).

## Results

Table 1 shows the overall picture of included studies. Sixteen studies were found that involved team sports; twelve of them focused on soccer, while the rest focused on cricket (Veness et al., 2017), basketball (Moreira et al., 2018), Australian football (Smith et al., 2015; Weerakkody et al., 2020), rugby (Smith et al., 2015), field hockey (Smith et al., 2015), and volleyball (Fortes et al., 2021a). Moreover, the majority of included studies recruited male athletes.

**Table 1:** Overview of publication details.

**Publication**	**Subjects**	**Level and specialization**	**Fatigue inducing task**	**Sport-specific motor performance**	**Type of assessment**	**Post-test MD**	**90% CI^a^ 95% CI^b^ ES [d]^c^ ES [$\eta_{p}^{\;2}$^d^**
Smith et al. (2015)	Male (*n =* 10; 22 ± 2 yr)	Well-trained soccer, Australian football, rugby and field hockey athletes	90 min AX-CPT	Physical performance	Intermittent running		
					Velocity (*ms*^− 1^) Total distance (m)	−0.04^*^ −91.00^*^	
Smith et al. (2016) Study 1	Male (*n =* 12; 24.0 ± 0.4)	Moderately trained soccer athletes	30 min Stroop task	Physical performance	Yo-Yo IR1		2.37 ^d^
					Total distance (m)	−207^***^	
Study 2	Male (*n =* 14; 19.6 ± 3.5)	Well-trained soccer athletes		Technical performance	Loughborough soccer passing test		
					Penalty time (s)	4.70^*^	0.76^d^
					Loughborough soccer shooting test		
					Point per shot (n)	−0.7.0^**^	0.75^d^
Badin et al. (2016)	Male (*n =* 20; 17.8 ± 1 yr)	Elite soccer athletes	30 min Stroop task	Physical performance	Small-sided game (5 vs. 5)		
					Total distance (m)	0.00	(−2.20, 2.00)^a^ 0.00^d^
				Technical performance	Involvement + (%)	−7.00^##^	(−16.00, −3.80)^a^ −0.73^d^
					Possessions + (%)	−8.00^##^	(−15.20, −3.60)^a^ −0.63^d^
Smith et al. (2017)	Male (*n =* 14; 19.6 ± 3.5 yr)	Well-trained soccer athletes	30 min Stroop task	Technical performance	Loughborough soccer passing test		
					Errors (n)	1.50^**^	(−2.60, −0.40)^b^ 0.39^d^
					Missed target (n)	8.00^*^	(−1.40, −0.10)^b^ 0.34^d^
Veness et al. (2017)	Male (*n =* 10; 21 ± 8 yr)	Elite cricket athletes	30 min Stroop task	Physical performance	Run-two test		
					Completion time (s)	0.10^**^	−0.51^c^
					Yo-Yo IR1		
					Total distance (m)	160.00^*^	0.39^c^
Moreira et al. (2018)	(*n =* 48; 15.2 ± 1.2 yr)	Elite basketball athletes	30 min Stroop task	Technical performance	Small-sided game (4 vs. 4)		
					Turnover (n)	NA^#^	0.71^c^
Coutinho et al. (2018)	(*n =* 10; (age = 13.7 ± 0.5 yr)	Amateur soccer athletes	30 min Stroop task	Physical performance	Small-sided game (GK ± 6 vs. 6± GK)		
					Total distance (m)	−16.50	
Kunrath et al. (2020)	Male (*n =* 18; 21.8 ± 2.5 yr)	Amateur soccer athletes	30 min Stroop task	Physical Performance	Small-sided game (GK ± 3 vs. 3 ± GK)	39.50^**^	(−53.73, −9.53)^b^
					Total distance (m)		
Trecroci et al. (2020)	(*n =* 9; 17.6 ± 0.5 yr)	Sub-elite soccer athletes	30 min Stroop task	Physical Performance	Small-sided game (4 vs. 4 ± W)		
					Total distance (m)	−101.30^*^	(−1.24, 0.83)^a^ −0.21^c^
				Technical performance	Passing accuracy (%)	−6.20^*^	(−1.16, 0.17)^a^ −0.49^c^
Filipas et al. (2020)	Male (*n =* 36; 13–18 yr)	Youth soccer athletes	30 min Stroop task	Physical performance	Yo-Yo IR1 Total distance		
					14	NA^*^ NA^*^ NA^*^	
					16		
					18		
				Technical performance	Loughborough soccer passing test Penalty time (s)		
					14	3.50	
					16	2.50	
					18	7.30^***^	
Weerakkody et al. (2020)	Male (*n =* 25; 23.8 ± 4.6 yr)	Amateur Australian football athletes	30 min Stroop task	Physical performance	AFL agility (s)	−0.05	−0.13^c^
					20 m sprint (s)	0.01	−0.05^c^
					MLch test (points)	0.36	0.10^c^
					Yo-Yo IR1	−142.4^*^	−0.45^c^
				Technical performance	Brad Johnson kicking test (points)	−2.64^*^	−0.40^c^
Soylu and Arslan (2021)	Male (*n =* 18; 19.1 ± 1.2 yr)	Amateur soccer athletes	30 min Stroop task	Technical performance	Small-sided game		
					2 vs. 2 Interception (n)	−0.83^*^	0.75^c^
					Successful tackle (n)	−0.61^*^	0.78^c^
Filipas et al. (2021)	Male (*n =* 19; 20 ± 3)	Amateur basketball athletes	30 min tactical basketball videos	Technical performance	Free throw (score)	−3.10^*^	0.40^c^
					Ball lost (n)	0.61^*^	0.65^c^
					3 vs. 3 Interception (n)	−0.61	
					Successful tackle (n)	−1.72	1.95^c^
					Ball lost (n)	2.11^*^	1.66^c^
					4 vs. 4 Interception (n)	−0.45	
					Successful tackle (n)	−0.11	
					Ball lost (n)	0.94^*^	0.88^c^
Fortes et al. (2021a)	Male (*n =* 24; 15.7 ± 0.6)	Elite volleyball athletes	30 min Smartphone	Physical Performance	Yo-Yo IR1		
					Individual distance	NA	(0.47, 0.60)^b^ 0.06^c^
					Countermovement Jump	NA	(0.41, 0.55)^b^ 0.04 ^c^
Ciocca et al. (2022)	(*n =* 10; 17.2 ± 0.9)	Sub-elite soccer athletes	30 min video-based tactical task	Physical Performance	Small-sided game Total distance (m)	−5.10	0.05^c^
				Technical performance	Passing accuracy (%)	8.40	0.95^c^
					Successful tackle (n)	0.50	0.20^c^
					Control errors (n)	0.60	0.46^c^
Soylu et al. (2022)	(*n =* 24; 15.9 ± 1.0 yr)	Youth soccer athletes	30 min Stroop task	Technical performance	Small-sided game		
					2 vs. 2 Successful pass (n)	−1.10	(−2.61, −4.81) 0.25^c^
					Ball lost (n)	1.30^*^	(−1.65, −0.92)^b^ 2.05^c^
					3 vs. 3 Successful pass (n)	−2.13	(−3.26, 7.51)^b^ 0.23^c^
					Ball lost (n)	0.08	(1.19, 1.97)^b^ 0.19^c^
					4 vs. 4 Successful pass (n)	−7.12^*^	(2.82, 11.42)^b^ 1.04^c^
					Ball lost (n)	0.79^*^	(−0.96, −0.61)^b^ 2.72^c^

* *p* ≤ 0.05;

** *p* ≤ 0.01;

*** *p* ≤ 0.001;

# likely negative;

## very likely negative at 90% confidence interval;

NA, not available; Post-test MD, Post-test mean differences between experimental and control groups; ES, effect size $\eta_{p\;}^{\;2}$; AX-CPT, AX-continuous performance test; Yo-Yo IR1, Yo-Yo intermittent recovery test, level 1; MLch test, Matthew Lloyd clean hands test; GK, goalkeeper; W, wildcard; a: 90% CI; b: 95% CI; c: ES [d]; d: ES [$\eta_{p\;}^{\;2}$].

### Mental fatigue inducting task

Thirty minutes Stroop task is predominant in all fatigue-inducing tasks (Table 1). Moreover, Le Mansec et al. (2017) requested athletes to complete AX-continuous tasks in 90 min. Filipas et al. (2021) and Ciocca et al. (2022) examined the 30 min tactical task in basketball and soccer, respectively. Finally, Fortes et al. (2021a) evaluated the influence when athletes expose to screens (e.g., cellphones) with social network apps for 30 min, which is more realistic in the modern environment.

### Sport-specific motor performance outcome

As shown in Table 1, eleven investigations examined physical performance, including 7 types of team sports; they are soccer (Smith et al., 2015, 2016; Badin et al., 2016; Coutinho et al., 2018; Filipas et al., 2020; Kunrath et al., 2020; Trecroci et al., 2020; Ciocca et al., 2022), cricket (Veness et al., 2017), Australian football (Weerakkody et al., 2020), and volleyball (Fortes et al., 2021a). Additionally, one study mixed 4 types of team sport athletes (soccer, Australian football, rugby, and field hockey) (Smith et al., 2015).

Furthermore, eleven experiments were done on technical performance in 3 types of team sports: soccer (Badin et al., 2016; Smith et al., 2016, 2017; Moreira et al., 2018; Filipas et al., 2020; Trecroci et al., 2020; Soylu and Arslan, 2021; Ciocca et al., 2022; Soylu et al., 2022), basketball (Filipas et al., 2021), and Australian football (Weerakkody et al., 2020).

#### Physical performance

Specifically, to examine the impact of MF among team sports athletes, Smith et al. (2015) employed a high-intensity, intermittent running regimen, which was highly relevant to the activity profile of intermittent team sports. The results showed that velocity (1.50 ± 0.18*ms*^−1^ vs. 1.54 ± 0.18 *ms*^−1^, *p* = 0.02), and total distance (4,072 ± 409 m vs. 4,163 ± 430 m, *p* = 0.02) were significantly different between the MF and control group (Smith et al., 2015). Consistently, Smith et al. (2016) study 1 corroborated the findings and showed a total distance of Yo-Yo intermittent recovery test, level 1 (Yo-Yo IR1) was dramatically impaired in the MF group compared with the control group (1,203 ± 402 m vs. 1,410 ± 354 m; *t*_11_ = 7.19, *P* < 0.001, ES = 2.37). Further, a similar finding was detected in cricket (1,732 ± 402 m vs. 1,892 ± 357 m; MF vs. control group, *p* = 0.023, ES = 0.39) (Veness et al., 2017), and Australian football athletes (1,040 ± 492.75 m vs. 1,182.40 ± 537.78 m; MF vs. control group, *p* = 0.03, ES = −0.45) (Weerakkody et al., 2020). Interestingly, a moderating effect of age was detected by Filipas et al. (2020) as older athletes showed greater declines in Yo-Yo IR1 distance.

To investigate more ecologically, however, the results showed inconsistency with running performance. The very first investigation was from Badin et al. (2016). The authors recruited a small-sided game (SSG) and did not uncover any significant difference between the experimental and control group in total distances running (1,531 ± 142 m vs. 1,531 ± 125 m, ES = 0.00). The non-significant finding was corroborated by another two studies that investigated total distance in SSG (*P* > 0.05) (Trecroci et al., 2020; Ciocca et al., 2022). Inconsistently, Kunrath et al. (2020) conducted a study to examine tactical and physical performance together. The result showed running performance measured as total distance significantly increased in the MF group (1,375.40 ± 1,050.00 m vs. 1,335.90 ± 94.30 m, *p* = 0.008, ES = 0.396), for the reduced accuracy in tactical actions (e.g., offensive and defensive coverage).

Moreover, Weerakkody et al. (2020) investigated other SSMP and did not find the negative impact of MF on Australian Football League agility (8.46 ± 0.40 s vs. 8.51 ± 0.32 s, *p* = 0.51, ES = −0.13), 20 m sprint (3.23 ± 0.15 s vs. 3.22 ± 0.30 s, *p* = 0.38, ES = −0.05), and Matthew Lloyd clean hands test (14.72 ± 3.69 vs. 14.36 ± 3.83, *p* = 0.66, ES = 0.10), when compared with the control group. Additionally, Fortes et al. (2021a) did not detect any significant effect of MF on endurance performance measured by the Yo-Yo test [*F*_(4, 20)_ = 0.05; *p* = 0.81; ES: trivial], and Countermovement Jump [*F*_(4, 20)_ = 0.01; *p* = 0.91; ES: small] induced by the social media using on smartphone before 4 weeks training sessions.

#### Technical performance

In the lab setting, MF significantly influenced SSMP, measured as increased penalty time (*t*_13_ = 2.93; *p* = 0.012; $\eta_{p}^{\;2}$ = 0.76) (Smith et al., 2016), the number of errors [*F*_(1, 13)_ = 8.19; *p* = 0.01; $\eta_{p}^{\;2}$ = 0.39] and missed target [*F*_(1, 13)_ = 6.67; *p* = 0.02; $\eta_{p}^{\;2}$ = 0.34] (Smith et al., 2017), as well as decreased point per shot (*t*_13_ = 3.24; *p* = 0.006; $\eta_{p}^{\;2}$ = 0.75) in the Loughborough soccer passing and shooting test. However, Filipas et al. (2020) found the adverse effect only exists in the U18 age group (increased penalty time: 15.3 ± 4.7 vs. 8.0 ± 3.1; *P* < 0.001) rather than U14 and U16 age groups.

Moreover, ball loss (n) in 2 vs. 2, 3 vs. 3, and 4 vs. 4 SSG were dramatically impaired in the condition of MF of soccer athletes (Soylu and Arslan, 2021; Soylu et al., 2022). In contrast, only interception (2.78 ± 1.17 vs. 3.61 ± 1.04; d = 0.75) and successful tackle (0.56 ± 0.62 vs. 1.17 ± 0.92; d = 0.78; *P* < 0.05) was influenced significantly which are more related to defensive skill in 2 vs. 2 SSG in soccer (Soylu and Arslan, 2021).

Besides soccer, basketball athletes showed a significant increase in turnover (ES = 0.71, 0.29;1.12) (Moreira et al., 2018) and a decrease in the free-throw score (*P* = 0.01) in SSG (Moreira et al., 2018). Moreover, Australian football athletes showed lower skill in kicking (18.32 + 5.27 vs. 20.96 + 6.35; *p* = 0.048) measured in Brad Johnson kicking test (Weerakkody et al., 2020).

### Psychophysiological outcome

Nineteen studies found significantly higher MF in experimental groups (see Table 2: Psychological Indicators). Notably, nine studies measured RPE in Table 2 and the result showed that RPE level was dramatically increased in mentally fatigued athletes (Smith et al., 2015, 2016; Badin et al., 2016; Coutinho et al., 2017; Veness et al., 2017; Trecroci et al., 2020; Filipas et al., 2021; Fortes et al., 2021a; Soylu et al., 2022). The variable of motivation also showed consistently non-significance in many studies. However, when athletes performed in SSG with different formats, the results showed a difference. That is, motivation was significantly lower in 2 vs. 2 and 3 vs. 3 (Soylu and Arslan, 2021). In contrast, it did not show any significance in the format of 4 vs. 4 (Soylu and Arslan, 2021).

**Table 2:** Psychophysiological outcomes of included publications.

**Publication**	**Physiological indicators**		**Psychological indicators**		
Smith et al. (2015)	Blood glucose ↔^*^	Blood lactate ↔^*^	MF↑^*^	RPE↑^*^	
	*VO*_2_↓^*^	Heart rate ↔^*^		Motivation ↔^*^	
Smith et al. (2016)	Heart rate ↔^*^		MF↑^*^	RPE↑^*^	
			ME↑^*^	Motivation ↔^*^	
Badin et al. (2016)	Heart rate↓^*^		MF↑^*^	RPE↑^*^	
				Motivation ↔^*^	
Smith et al. (2017)			MF↑^*^	Motivation ↔^*^	
			ME↑^*^		
Veness et al. (2017)			MF↑^*^	RPE↑^*^	
				Motivation ↔^*^	
Moreira et al. (2018)	Testosterone (Exp: ↔; Con: ↑^##^)		MF↑^*^		
	Alpha-Amylase (Exp: ↑^#^; Con: ↑^##^)				
	Cortisol (Exp: ↔; Con ↔)				
Coutinho et al. (2018)			MF↑^*^	RPE↑^*^	
Kunrath et al. (2020)			MF↑^*^	VF↑^**^	TD ↔^**^
				RT ↔^**^	OT ↔^**^
Trecroci et al. (2020)			MF↑^*^	RPE↑^***^	
	ME↑^***^	Motivation ↔^*^	
Filipas et al. (2020)			MF↑^*^	Motivation ↔^*^	
Weerakkody et al. (2020)			MF↑^*^	Motivation ↔^*^	
Filipas et al. (2021)			MF↑^*^	Motivation ↔^*^	
Soylu and Arslan (2021)			2 vs. 2		
			MF↑^*^	Feeling↑^*^	
			Felt Arousal↑^*^	Motivation ↓^*^	
			Cognitive demand↓^*^		
			3 vs. 3		
			MF↑^*^	Feeling↑^*^	
			Felt Arousal↑^*^	Motivation ↓^*^	
			Cognitive demand↓^*^		
			4 vs. 4		
			MF ↔^*^	Feeling↑^*^	
			Felt Arousal↑^*^	Motivation ↔^*^	
			Cognitive demand↓^*^		
Fortes et al. (2021a)			MF↑^***^	RPE↑^*^	
Ciocca et al. (2022)			RPE ↔^*^	Motivation ↔^*^	
Soylu et al. (2022)	2 vs. 2				
	Heart rate↑^**^	HR_max_↑^**^	MF↑^*^	RPE↑^*^	
			ME↑^*^	PACES ↔^*^	
	3 vs. 3				
	Heart rate↑^**^	HR_max_↑^**^	MF↑^*^	RPE↑^*^	
			ME↑^*^	PACES ↔^*^	
	4 vs. 4				
	Heart rate↑^**^	HR_max_↑^**^	MF↑^*^	RPE↑^*^	
			ME↑^*^	PACES ↔^*^	

MF, mental fatigue; ME, mental effort; RPE, the rating of perception effort; PP, peripheral perception; Exp, experimental group; Con, control group;

## Large and clear difference between time-points;

# Moderate and clear difference between time-points;

* significance between conditions;

** significance between time-points;

*** significant interaction;

VF, visual field; TD, tracking deviation; RT, reaction time; OT, number of omitted reactions; HR max, percentage of maximum heart rate; PACES, physical activity enjoyment scale. Higher↑, lower↓, no significant difference ↔ in the mental fatigue condition compared with control condition.

The traditional physiological indicator for fatigue such as blood glucose (*p* = 0.935) and blood lactate (*p* = 0.809) was also not influenced by MF (Smith et al., 2015). Regarding the parameter of heart rate, the included studies showed inconsistent results. Specifically, Smith et al. (2015); Badin et al. (2016), and Smith et al. (2016) showed that MF did not influence heart rate (*P* > 0.05), while Soylu et al. (2022) detected a significantly higher value of heart rate in 3 different formats of SSG. Furthermore, Moreira et al. (2018) found that the salivary parameters of alpha-amylase were considerably higher in mentally fatigued athletes (*P* < 0.05) after the performance task (e.g., basketball SSG).

## Discussion

This scoping review aims to summarize the present state of knowledge about the impact of the mental effect on the sport-specific motor, including physical and technical performance in team sport athletes. Moreover, to summarize the psychophysiological outcomes from the perspective of potential mechanisms regarding the effect of MF and understand existing theories related to performance reduction in team sports.

### Overview of team sports

Unsurprisingly, all these team sports (soccer, basketball, volleyball, and Australian football) have centered on ball sports, as the cognitive demands of ball sports are particularly demanding (Faubert and Sidebottom, 2012; Heppe et al., 2016). Athletes' movements and the ball's trajectories are often erratic in sports due to factors including interruptions, which can cause abrupt changes in course, and occlusions and segmentation, in which various objects obscure or disappear from view. As a result, those who compete in ball sports need to maintain vigilance the entire time (Faubert and Sidebottom, 2012; Smith et al., 2018), which raises the likelihood of MF.

Soccer significantly outnumbered other sports. Specifically, eleven investigations examined the effect of MF on soccer athletes, while the other four focused on basketball (Moreira et al., 2018; Filipas et al., 2021), cricket (Veness et al., 2017), volleyball (Fortes et al., 2021a), and Australian football (Weerakkody et al., 2020).

### The effect of mental fatigue on sport-specific motor performance

#### Physical performance

To our best knowledge, this is the first review to synthesize sport-specific motor performance, including physical and technical performance in team sports.

The different demands of physical performance in team sports are highlighted in the current review, which may cause different effects of MF. Specifically, physical performance such as intermittent endurance was impaired after the elicitation of acute MF in soccer (Smith et al., 2015, 2016; Filipas et al., 2020; Kunrath et al., 2020), cricket (Veness et al., 2017), and Australian football athletes (Weerakkody et al., 2020). In contrast, volleyball athletes were not influenced (Fortes et al., 2021a) by the difference in demand for low aerobic endurance for competition activities lasting less than 10 seconds, as there are typically 15-sec rest intervals between successive points (Freitas et al., 2014). The same result also showed in individual sports with a short duration of a performance task. For example, MF did not influence 100 m and 200 m dash performance in sprinters (Fortes et al., 2021b). Future studies may compare different types of sports (e.g., team and individual) to understand more about the mechanism of the effect of MF.

Moreover, the major attention on soccer athletes may be owing to the fact that these athletes may be more sensitive to MF than athletes in other sports as a result of their prolonged exposure to cognitive demands. It is also consistent with the definition of MF, which comes from “prolonged” cognitive tasks.

Therefore, adverse effects of MF may not extend to physical performance among all the team athletes. The effect is highly dependent on the action demands and the duration of the task or competition. However, more studies are required to directly compare these effects in different team sports.

To investigate more ecologically, Badin et al. (2016) tested total distance during possession 5 vs. 5 SSG, however, no impairment was detected in the MF condition. This non-significance was also reported in the goalkeeper + 6 vs. 6 + goalkeeper SSG (Coutinho et al., 2017). Inconsistently, the impairment of intermittent was shown in goalkeeper + 3 vs. 3 + goalkeeper SSG (Kunrath et al., 2020), and 4 vs. 4 + wildcard SSG (Trecroci et al., 2020).

Notably, the formats of SSG determine different responses in team-sport athletes. For example, maximum velocity, maximum acceleration, and deceleration increased with the decrease in the number of athletes (Gaudino et al., 2014; Silva H. et al., 2022). And some rules such as the inclusion or exclusion of goalkeepers had a large effect on physical load (Santos et al., 2021).

Since another recent review indicated that the impairment of performance during SSG could not be conclusive (Costa et al., 2022), there are several questions raised, for example, could the non-significant effect of MF on physical performance measured as intermittent endurance be from the less demand in the possession format of SSG in the study of Badin et al. (2016)? Are the number of athletes on each side of SSG in the study of Coutinho et al. (2017) too big to induce the adverse effect?

Previous studies only noticed a likely explanation of such contradictory findings of physical performance in SSG may be attributed to the different assessments. Indeed, Trecroci et al. (2020) considered accelerations >2 *m*/*s*^2^, while Badin et al. (2016) considered accelerations >2.78 *m*/*s*^2^. On the other hand, future studies could consider more about that MF may influence differently in a variety of SSG formats.

#### Technical performance

Regarding technical performance, the results showed rather a consistency in the impairment in team sports, including soccer (Badin et al., 2016; Smith et al., 2016, 2017; Filipas et al., 2020; Trecroci et al., 2020), basketball (Moreira et al., 2018; Filipas et al., 2021; Soylu et al., 2022), and Australian football (Weerakkody et al., 2020). A most recent study conducted by Sun et al. (2022a) synthesized the adverse effect of MF on soccer players' technical performance. The authors found the adverse effects of defensive technique are more than that of offensive technique. However, the current review showed impairment in both types of technical performance (see Table 1) and could not compare the specific effect on defensive and offensive technique due to it being out of scope. It is considered important to examine different types (defensive and offensive) of techniques in all team sports in the future.

### Mechanism of mental fatigue in team sport

Previous studies measured psychobiological responses shown in Table 2 and provide a way to understand the mechanism of MF in a team sport.

The self-paced nature of exercise largely exists in team sports and it could be used by athletes to mitigate acute fatigue in matches (Aughey, 2010), including MF. A “central controller” (central nervous system) is thought to determine exercise intensity in response to various physiological signals and regulates this intensity to avoid premature fatigue (Noakes et al., 2005; Noakes, 2012). Therefore, it is reasonable that previous studies shown in Table 2 did not detect a higher level of these physiological indicators such as blood lactate (Smith et al., 2015) and heart rate (Smith et al., 2015, 2016; Badin et al., 2016) in MF condition. The results could be explained by the study of Taheri and Irandoust (2020). The authors indicated that low-intensity exercise alters brain structure and function from cells and molecules to behavior and system, which may reduce fatigue due to better cognition. Therefore, more studies are required to examine the effect of the self-paced nature of low-intensity exercise in team sports on MF. On the other hand, although Soylu et al. (2022) detected a higher level of heart rate between time points (pre and post-test) in MF conditions, it is not conclusive due to the comparison between conditions and interaction should be focused more.

During exercise in a team sport, homeostasis threats are signaled *via* afferent feedback (Noakes, 2012). This perceived magnitude of homeostatic threats is generated as a higher level of RPE in the previous studies (see Table 2: RPE). Moreover, Soylu et al. (2021) argued that RPE increased due to MF lowering performance profile and shifting perceived exertion against exhaustion time. Consistent with the psychobiological model proposed by Marcora (2008), explaining the negative effects of MF on self-paced exercise such as team sports in physical performance.

Therefore, the current review indicates that the psychobiological model of exercise performance could be the prestigious theory used in a team sport, rather than the traditional “catastrophe” theory to explain the impacts of MF.

Moreover, the potential extension of the model should be noticed. The reductions in technical performance were not attributed to the raised level of RPE (Sun et al., 2022c). It could be confirmed in team sports due to the result shown in Table 2. Specifically, four studies investigated the technique of sport-specific motor performance in soccer (Smith et al., 2017; Soylu and Arslan, 2021; Soylu et al., 2022), and basketball (Moreira et al., 2018) did not report RPE. Thus, it is necessary to divide the investigation of the sport-specific motor performance to be two parts; physical and technical performance due to the different mechanisms of the effects.

Notably, Kunrath et al. (2020) detected decreased vision perceptual in mentally fatigued athletes, which could confirm that besides RPE, attention could be a crucial factor attributed to the impairment of performance in team sport athletes (e.g., soccer). Consistently, the attention resources could be manipulated to combat MF and increase subsequent performance significantly (Sun et al., 2022b,c,e).

Therefore, the current review recognizes the psychological model of exercise performance (Marcora, 2008), and its potential extension (Sun et al., 2022d) as the best theory for team sports due to the consistency of the mechanism with the impacts of MF on sport-specific motor performance.

## Limitations

There are a few significant caveats to this review. First, the systematic review focuses only on SSMP, defined as physical and technical performance, excluding perceptual-cognitive skills in team sports. Future research could examine all three of these skilled performances together to acquire more complete data, as they are interconnected and all contribute to athletes' performance, especially when the sports are open-skill (Soylu et al., 2021; Dong et al., 2022). Also, MF impaired athletes' perceptual-cognitive skills such as decision-making due to the inability of perceiving environmental clues with limited attention resources (Gantois et al., 2019; Fortes et al., 2020, 2022). The overall picture of athletes' performance with SSMP and perceptual-cognitive skills needs to be examined.

Second, the current review only detected the inconclusive data in different formats of SSGs (e.g., number differences, with or without a goalkeeper). Perhaps the effect may be larger when there are fewer athletes and a goalkeeper in the SSGs due to the increased physical load (Santos et al., 2021; Silva H. et al., 2022). However, it is necessary to be examined by future studies.

Furthermore, it was suggested that MF influence more offensive skill due to cognitive resources being more exhausted (Sun et al., 2021). To contrast, the suggestion was not supported by a recent meta-analysis that showed a larger effect on defensive skills (Sun et al., 2022a). The current review only creates the overall picture of technical skill. Future studies should clarify the conflicting result.

Finally, selecting only English-language studies may further restrict the representation of the findings.

## Conclusion

In team sports, such as soccer, cricket, Australian football, and basketball, MF adversely influences sport-specific motor performance, including physical and technical performance. Future studies may examine different formats of SSG (e.g., the number of playing athletes, with or without wildcard and goalkeeper) due to different responses and effects of subsequent performance in mentally fatigued athletes. Moreover, the most relevant theory should be the psychological model of exercise performance and its potential extension of attention resources regarding the topic of the impacts of MF on performance among athletes in team sports.

## Data availability statement

The original contributions presented in the study are included in the article/Supplementary material, further inquiries can be directed to the corresponding author.

## Author contributions

All authors listed have made a substantial, direct, and intellectual contribution to the work and approved it for publication.
