# Supplementary material for: The effects of mental fatigue on sport-specific motor performance among team sport athletes: A systematic scoping review
Source: Front Psychol. 2023 Apr 11;14:1143618. doi: 10.3389/fpsyg.2023.1143618 (PMC10128192; doi:10.3389/fpsyg.2023.1143618)
Supplement: Supplementary file 1 [file Table_1.doc]

**Table S1 - Detailed search strategy**

| **Database** | **Search Keywords** | **Results** |
| --- | --- | --- |
| **PubMed**  **(- May 2022)** | 1. "mental fatigue"[Mesh] OR “mental fatigue” OR “mental exertion” OR “cognitive fatigue” OR “cognitive exertion” OR “mental exhaustion” OR “mental tiredness” **4, 4547** 2. sport, **396, 383** 3. (1) AND (2), **433** 4. Limited to “Clinical Trial” and “Randomized Controlled Trial”, **72** | 72 |
| **Web of Science**  **(- May 2022)** | 1. TS=(“mental fatigue” OR “mental exertion” OR “cognitive fatigue” OR “cognitive exertion” OR “mental exhaustion” OR “mental tiredness”), **6, 465** 2. TS=(“technical performance” OR “technique” OR “skill” OR “physical performance” OR “endurance” OR “power” OR “performance”), **23, 346,665** 3. TS=(sport), **574, 469** 4. (1) AND (2), **349** 5. Limited to articles, **289** 6. Limited to human, **191** | 191 |
| **EBSCOhost**  **(- May 2022)** | 1. TI ( “mental fatigue” OR “mental exertion” OR “cognitive fatigue” OR “cognitive exertion” OR “mental exhaustion” OR “mental tiredness” ), **35** 2. sport, **64, 247** 3. (1) AND (2), **23** | 23 |
| **Scopus**  **(- May 2022)** | TITLE-ABS-KEY ( "mental fatigue"  OR  "mental exertion"  OR  "cognitive fatigue"  OR  "cognitive exertion"  OR  "mental exhaustion"  OR  "mental tiredness" ), 4, 532TITLE-ABS-KEY ( sport ), 291, 518  1. (1) AND (2), **200** 2. Limited to articles, **155** 3. Limited to English, **142** | 142 |
