# Supplementary material for: The effects of mental fatigue on sport-specific motor performance among team sport athletes: A systematic scoping review
Source: Front Psychol. 2023 Apr 11;14:1143618. doi: 10.3389/fpsyg.2023.1143618 (PMC10128192; doi:10.3389/fpsyg.2023.1143618)
Supplement: Supplementary file 2 [file Table_2.docx]

**Table S2 “Qualsyst” of Quality Assessment**

| Publication | Question/objective described | Appropriate study design | Appropriate subject selection | Characteristics sufficiently described | Random allocation | Researchers blinded | Subjects blinded | Outcome measures well defined and robust to bias | Appropriate sample size | Analytic methods well described | Estimate of variance reported | Controlled for confounding | Results reported in detail | Conclusion supported by results? | Rating |
| --- | --- | --- | --- | --- | --- | --- | --- | --- | --- | --- | --- | --- | --- | --- | --- |
| Smith et al. (2015) | 2 | 2 | 2 | 2 | NA | 0 | 1 | 2 | 1 | 1 | 2 | 0 | 2 | 2 | medium |
| Smith et al. (2016) | 2 | 2 | 2 | 2 | NA | 0 | 1 | 2 | 1 | 2 | 2 | 1 | 2 | 2 | high |
| Badin et al. (2016) | 1 | 2 | 2 | 1 | NA | 1 | 0 | 2 | 1 | 2 | 2 | 1 | 1 | 2 | medium |
| Smith et al. (2017) | 2 | 2 | 2 | 1 | NA | 2 | 1 | 2 | 1 | 2 | 2 | 1 | 1 | 2 | medium |
| Veness et al. (2017) | 2 | 2 | 2 | 2 | NA | 0 | 1 | 2 | 2 | 2 | 2 | 0 | 2 | 2 | high |
| Coutinho et al. (2017) | 2 | 2 | 2 | 2 | NA | 0 | 0 | 2 | 2 | 2 | 2 | 0 | 2 | 2 | medium |
| Moreira et al. (2018) | 2 | 2 | 2 | 2 | NA | 0 | 1 | 2 | 2 | 2 | 1 | 2 | 2 | 2 | high |
| Coutinho et al. (2018) | 2 | 2 | 2 | 2 | 1 | 0 | 0 | 2 | 2 | 2 | 2 | 0 | 2 | 2 | high |
| Kunrath et al. (2018) | 1 | 1 | 2 | 1 | 1 | 0 | 0 | 2 | 0 | 2 | 2 | 0 | 1 | 2 | low |
| Kunrath et al. (2020) | 2 | 2 | 2 | 2 | 1 | 0 | 0 | 2 | 1 | 2 | 2 | 0 | 2 | 2 | medium |
| Trecroci et al. (2020) | 2 | 2 | 2 | 2 | NA | 0 | 0 | 2 | 1 | 2 | 2 | 0 | 2 | 2 | medium |
| Filipas et al. (2020) | 2 | 2 | 2 | 2 | NA | 2 | 0 | 1 | 2 | 2 | 0 | 1 | 1 | 1 | medium |
| Bahrami et al. (2020) | 1 | 0 | 1 | 1 | 1 | 0 | 0 | 1 | 1 | 0 | 2 | 0 | 0 | 1 | low |
| Weerakkody et al. (2020) | 2 | 2 | 2 | 2 | NA | 0 | 0 | 2 | 2 | 2 | 2 | 0 | 2 | 2 | medium |
| Filipas et al. (2021) | 2 | 2 | 2 | 2 | NA | 0 | 2 | 2 | 1 | 2 | 2 | 0 | 2 | 2 | high |
| Soylu and Arslan (2021) | 2 | 2 | 1 | 2 | NA | 0 | 0 | 2 | 1 | 2 | 2 | 0 | 2 | 2 | medium |
| Fortes et al. (2021) | 2 | 2 | 2 | 2 | 2 | 2 | 0 | 2 | 1 | 2 | 2 | 1 | 2 | 2 | high |
| Ciocca et al. (2022) | 2 | 2 | 1 | 2 | 2 | 0 | 0 | 2 | 1 | 2 | 2 | 0 | 2 | 2 | medium |
| Soylu et al. (2022) | 2 | 2 | 1 | 2 | NA | 0 | 0 | 2 | 1 | 2 | 2 | 0 | 2 | 2 | medium |

NA: not applicable, 2 indicates yes, 1 indicates partial, 0 indicates no Quality; Quality score: $\geq$ 75% high, 55% -75% medium, $\leq$ 55% low.
